# Brain morphometry in 22q11.2 deletion syndrome: an exploration of differences in cortical thickness, surface area, and their contribution to cortical volume

**DOI:** 10.1038/s41598-020-75811-1

**Published:** 2020-11-02

**Authors:** M. Gudbrandsen, E. Daly, C. M. Murphy, C. E. Blackmore, M. Rogdaki, C. Mann, A. Bletsch, L. Kushan, C. E. Bearden, D. G. M. Murphy, M. C. Craig, Christine Ecker

**Affiliations:** 1 Department of Forensic and Neurodevelopmental Sciences, and the Sackler Institute for Translational Neurodevelopmental Sciences, Institute of Psychiatry, Psychology and Neuroscience, King’s College, London, UK; 2 Behavioural Genetics Clinic, Adult Autism and ADHD Services, Behavioural and Developmental Clinical Academic Group, South London and Maudsley Foundation, NHS, London, UK; 3 Department of Psychosis Studies, Institute of Psychiatry, Psychology and Neuroscience, King’s College, London, UK; 4 Department of Child and Adolescent Psychiatry, Institute of Psychiatry, Psychology and Neuroscience, King’s College, London, UK; 5 Department of Child and Adolescent Psychiatry, Psychosomatics and Psychotherapy, University Hospital, Goethe University, Frankfurt, Germany; 6 Department of Psychiatry and Biobehavioral Sciences, Semel Institute for Neuroscience and Human Behavior, University of California-Los Angeles, Los Angeles, CA USA; 7 Department of Psychology, University of California-Los Angeles, Los Angeles, CA USA; 8 National Autism Unit, Bethlem Royal Hospital, London, UK

**Keywords:** Molecular neuroscience, Magnetic resonance imaging

## Abstract

22q11.2 Deletion Syndrome (22q11.2DS) is the most common microdeletion in humans, with a heterogenous clinical presentation including medical, behavioural and psychiatric conditions. Previous neuroimaging studies examining the neuroanatomical underpinnings of 22q11.2DS show alterations in cortical volume (CV), cortical thickness (CT) and surface area (SA). The aim of this study was to identify (1) the spatially distributed networks of differences in CT and SA in 22q11.2DS compared to controls, (2) their unique and spatial overlap, as well as (3) their relative contribution to observed differences in CV. Structural MRI scans were obtained from 62 individuals with 22q11.2DS and 57 age-and-gender-matched controls (aged 6–31). Using FreeSurfer, we examined differences in vertex-wise estimates of CV, CT and SA at each vertex, and compared the frequencies of vertices with a unique or overlapping difference for each morphometric feature. Our findings indicate that CT and SA make both common and unique contributions to volumetric differences in 22q11.2DS, and in some areas, their strong opposite effects mask differences in CV. By identifying the neuroanatomic variability in 22q11.2DS, and the separate contributions of CT and SA, we can start exploring the shared and distinct mechanisms that mediate neuropsychiatric symptoms across disorders, e.g. 22q11.2DS-related ASD and/or psychosis/schizophrenia.

## Introduction

22q11.2 Deletion Syndrome (22q11.2DS), also known as DiGeorge or Velo-Cardio-Facial syndrome, is the most common microdeletion syndrome in humans with an estimated prevalence of 1 in 4000^1^. The phenotypic consequences of the microdeletion are both complex and highly variable across the lifespan^2^, and encompass a broad range of medical and neuropsychiatric conditions. Specifically, 22q11.2DS is widely associated with cardiac and palatal abnormalities, developmental delay and cognitive deficits, as well as neuropsychiatric disorders including schizophrenia and autism spectrum disorder^2^. As such, the heterogeneity of 22q11.2DS has widely been used as a genetic model for a variety of medical conditions, as well as for psychiatric and developmental disorders^2^.

To date, several structural neuroimaging studies have examined the neuroanatomical underpinnings of the 22q11.2 microdeletion. For example, previous studies consistently report significant reductions in total intracranial brain volume in individuals with 22q11.2DS compared to typically developing (TD) controls^3^. These global differences in brain volume appear to be driven by commensurate reductions in both grey and white matter volume across both hemispheres with occipital lobes being the most affected, and frontal lobes being the least affected^3^. However, measures of total cerebral brain volume are relatively unspecified as cortical volume is a product of cortical thickness (CT) and surface area (SA), both of which have distinct developmental trajectories and relate to distinct aspects of the cortical architecture^4^. A number of recent studies have indeed explored the neuroanatomy of 22q11.2DS by focusing on CT and SA in addition to cortical volume (CV). This is of importance as both of these features are believed to represent independent (i.e. uncorrelated) sources of neuroanatomical variability, and contribute differentially to differences in regional cortical volume^5^. Studies examining CT and SA in 22q11.2DS, including a recent large-scale study from the ENIGMA 22q11.2DS consortium, typically report significantly increased thickness across the cortex including bilateral frontal lobe, insula, inferior parietal lobe and bilateral posterior medial cortex^6–8^. In contrast, there are significant reductions in SA, particularly across frontal lobes, superior parietal lobes, medial occipital lobes and anterior cingulate regions^6–8^. It has further been suggested that spatial differences observed in CT appear to overlap with that of SA in some parts of the brain^7^, and that the differences in total cortical grey matter volume in 22q11.2DS may be driven by reduced SA rather than increased CT^6,8^. Moreover, previous studies suggest that the relative ‘sparing’ of the frontal lobes may be related to the combined effect of differential variability in CT and SA (i.e. increased CT and decreased SA), which together may mask a significant difference in C^8,9^. However, no study to date has quantified the degree of spatial overlap between differences in CT and SA directly in 22q11.2DS, nor identified their contribution to regional differences in CV. This is important as it will allow us to further disentangle the complex neuroanatomy of the condition, which may have a differential impact on the heterogeneous clinical presentation. A vertex-wise approach will be used to examine vertices across the entire cortical surface in order to identify clusters with significant between-group differences spanning across several brain regions. This is particularly useful when examining complex and heterogeneous neurodevelopmental disorders, such as 22q11.2DS, where structural alterations have been reported to be spatially distributed, affecting several large-scale neurocognitive networks^10^.

The aim of the present study was to investigate regional differences in CV, SA, and CT—as well as their relationship—in individuals with 22q11.2DS compared to typically developing controls—using a vertex-based approach. This approach allowed us to identify: (1) the spatially distributed networks of differences in CT and SA; (2) the degree of spatial overlap between them; and (3) their relative contribution to observed differences in regional CV. Given recent evidence^7,11^, it was expected that the spatial distribution of differences in CT and SA significantly varies across the cortex between individuals with the 22q11.2 microdeletion and TD controls, and that the vertex-wise overlap of between-group differences in CT and SA exerts a significant effect on measures of CV in some areas of the cortex (e.g. the frontal lobes).

## Materials and methods

### Participants

Sixty-two individuals with a confirmed molecular diagnosis of 22q11.2DS (6–31 years, 30 male and 32 female) and fifty-seven typically developing controls (6–27 years, 27 males and 30 females) were recruited at two sites: (1) The Institute of Psychiatry, Psychology and Neuroscience (IoPPN), London, UK; and (2) The Semel Institute for Neuroscience and Human Behaviour, University of California (UCLA), Los Angeles, US. Approximately equal proportions of cases were recruited at each site (IoP N = 28, UCLA N = 34) (see Table 1 for participant demographics). These participants were previously published on by our group^11^. The deletion was confirmed using by in-situ hybridization (FISH) or microarray. Exclusion criteria for all participants included contraindications to MRI and medical conditions or chromosomal anomaly other than 22q11.2DS, which may be associated with autism spectrum disorder (ASD) or psychosis (e.g. tuberous sclerosis, Fragile X syndrome or Prader-Willi syndrome). However, individuals with ASD, attention deficit hyperactivity disorder (ADHD), obsessive compulsive disorder (OCD), psychosis, depression or anxiety were included, as these are common co-morbid features of 22q11.2DS. All 22q11.2DS individuals were assessed for prodromal symptoms of psychosis using the Structured Interview for Prodromal Syndromes (SIPS)^12^. Here, the existence and severity of prodromal positive psychotic symptoms (e.g. unusual thought content, suspiciousness, grandiose ideas), and negative psychotic symptoms (e.g. social anhedonia, avolition, expression of emotion) were rated on a scale from 0–6 (0 = absent; 6 = extreme severe level). Any individual who scored between 3–5 on positive psychotic symptoms were rated as exhibiting prodromal symptoms of psychosis, and those who scored 6, were rated as exhibiting psychotic symptoms. This resulted in 2 individuals who met criteria for ‘psychotic symptoms’, and in 9 individuals who met criteria for ‘prodromal symptoms of psychosis’.

**Table 1:** Participant demographics and global brain measures for 22q11.2DS compared to controls.

	22q11.2DS		Control		Significance	
	(n = 62 [30♂, 32♀] )		(n = 57 [27♂, 30♀])		*t*(117)	*p*
Age, years	16 ± 7	(6–31)	15 ± 6	(6–27)	0.71	0.48
Full-scale IQ	82 ± 13	(60–116)	114 ± 16	(76–148)	− 11.84	< 0.001
Total Grey Vol (L)	0.59 ± 0.23	(0.41–0.89)	0.64 ± 0.24	(0.41–0.89)	− 1.29	0.201
Average CT (mm)	2.72 ± 0.14	(2.59–3.06)	2.73 ± 0.12	(2.46–2.99)	− 0.64	0.522
Total SA (m^2^)	0.20 ± 0.20	(0.13–0.25)	0.23 ± 0.20	(0.19–0.27)	− 5.95	< 0.001
Psychotropic medication (none/antipsych/antidep/stimul)	(43/5/10/4)		(49/0/6/2)			

Data expressed as mean ± standard deviation (range). Medication: none: no medication; antipsych: antipsychotic medication; antidep: antidepressant medication; stimul: psychostimulant medication.

Due to the complex mental and physical health profile of individuals with 22q11.2DS, it is difficult to identify an appropriate control group and we therefore included both siblings who shared a similar psychosocial background, as well as unrelated controls. Of the n = 119 included individuals, there were 7 controls who had a sibling with 22q11.2DS participating in the study, one individual with 22q11.2DS who had two siblings without the microdeletion, one 22q11.2DS sibling pair, as well as 6 control sibling pairs.

Overall intellectual ability was assessed using the Wechsler Abbreviated Scale of Intelligence (WASI-^13^). Participants with a full-scale IQ below 60 were excluded from the study.

All participants, and accompanying parents/guardians for those under 18, gave informed written informed consent in accordance with ethics approval by National Research Ethics Service (NRES) Committee South Central (study reference: 12/SC/0576) and/or the UCLA Institutional Review Board (IRB). These were all in accordance with the Declaration of Helsinki.

### MRI data acquisition

All participants were scanned with a contemporary MRI scanner operating at 3 T (Signa; GE Medical Systems at the IoPPN, London; Siemens “Tim Trio” at UCLA). High-resolution structural T1-weighted volumetric images were acquired with full head coverage, 166 contiguous slices (1.2-mm thickness, with 1.2 × 1.2-mm in-plane resolution), repetition time/echo time (TR/TE) of 7/2.8 ms (flip angle = 8in, FOV = 26 cm). Parameters were matched across sites to obtain images with similar contrast and voxel sizes and consistent image quality was ensured by a semi-automated quality control procedure at both sites. These methods were previously published by our group^11^.

### Cortical surface reconstruction using FreeSurfer

FreeSurfer v6.0.0 software (https://surfer.nmr.mgh.harvard.edu/) was used to derive models of the cortical surface for each T_1_-weighted image. These well-validated and fully automated procedures have been extensively described elsewhere^14–18^. In brief, a single filled white-matter volume was generated for each hemisphere after intensity normalization, extra-cerebral tissue was cropped, and image segmentation performed using a connected components algorithm. A triangular tessellated surface was then generated for each white-matter volume by fitting a deformable template, resulting in a cortical mesh for the pial (i.e. outer) and white-matter (i.e. inner) surface. The resulting surface models were visually inspected for reconstruction errors, and either (1) accepted ‘as is’, (2) rejected, mostly due to existence of severe movement artefacts, or (3) referred for manual editing. Manual edits were performed by making changes to the pial (i.e. grey matter) outline, to the white matter outline, or both. Following manual editing, images were (re-)pre-processed and re-assessed for reconstruction errors. Further details on quality assessments and scan exclusion rates are described in our previously published study^11^. Measures of CT were computed as the closest distance from the grey-white matter boundary to the grey matter-cerebrospinal fluid boundary at each vertex on the tessellated surface^16^. For each participant, we also computed mean cortical thickness across the entire brain and total surface area. Vertex-based estimates of SA were derived as outlined by Ref.^19^. To improve the ability to detect population changes, each parameter was smoothed using a 10-mm surface-based smoothing kernel.

### Statistical analyses

Statistical analysis was conducted using the SurfStat toolbox (https://www.math.mcgill.ca/keith/surfstat/) for Matlab (R2019a; MathWorks). To first determine developmental trajectories of CV, CT and SA, we tested for linear, quadratic, and cubic effects of age, in addition to the main effect of group in a vertex-wise fashion. Here, an F-test for nested model comparisons was performed at each vertex, employing a step-up model selection procedure. Initially, the linear (i.e. most reduced) model was compared to a more complex (i.e. quadratic) model in order to determine if the addition of a quadratic age effect significantly improved the goodness-of-fit. If the quadratic model performed significantly better, it was then compared to the most complex (i.e. cubic) model, which contained a linear, quadratic, and cubic age term. Corrections for multiple comparisons across the whole brain were performed using random-field theory (RFT)-based cluster-corrected analysis for non-isotropic images using a *p* < 0.05 (two-tailed) cluster-significance threshold^20^. This procedure allowed us to identify the most parsimonious model at each vertex, i.e. the simplest plausible model that explained age-related variability across all measures (i.e. CV, CT and SA), with the smallest set of predictors. All nested model comparisons were performed based on the combined sample of 22q11.2DS and TD individuals. Based on the nested model comparison, we established that the quadratic model provided a significantly better fit than the linear model in several clusters across the cortex (see Supplementary Information S1). The quadratic model was subsequently used to examine between-group differences.

More specifically, we examined between-group differences and age-by-group interactions by applying a general linear regression model (GLM) at each vertex *i* and subject *j*, with (1) site, group, gender, site, and relatedness (encoded as ‘yes’ or ‘no’) as categorical fixed-effects factor; and (2) linear and quadratic terms for age, as well as their interactions with group. As 22q11.2DS, compared to controls, had decreased overall surface area, this was added as a co-variate so that:

$$ Y_{i} = \beta_{0} + \beta_{1} Site_{j} + \beta_{2} Group_{j } + \beta_{3} Age_{j } + \beta_{4} Age^{2}_{j} + \beta_{5} \left( {AgexGroup} \right) + \beta_{6} \left( {Age^{2} xGroup} \right) + \beta_{7} Gender + \beta_{8} IQ_{j} + \beta_{9} Relatedness_{j} + \beta_{10} TotalSA_{j} + \varepsilon_{j} , $$

where *ε*_*i*_ is the residual error at vertex *i*. All included continuous covariates were mean centered across groups to improve interpretability of the coefficients. Corrections for multiple comparisons across the whole brain were performed using ‘random field theory’ (RFT)-based cluster analysis for non-isotropic images using a cluster based significance threshold of *p* < 0.001, 0.01, and 0.05 (two-tailed)^20^ to examine the robustness of our results across cluster thresholds. All statistical effects (i.e. between-group differences and age-by-group interactions) were mapped onto the FreeSurfer high-resolution common-group template in standard space (i.e. ‘fsaverage’ with ~ 300,000 vertices). To rule out potential effects of psychosis, a subgroup analysis was also performed within the 22q11.2DS groups to establish the effects of prodromal symptoms of psychosis. Moreover, to examine potential associations between group and IQ, *Pearson’s r* correlations between IQ and neuroanatomical measures CV, CT and SA were examined.

To compare frequencies of unique or overlapping differences in each morphometric parameter, the resulting spatially-distributed binary patterns of differences unique to CT and/or SA, as well as their overlap regardless of the sign (i.e. based on their statistical threshold), were then compared using a *χ*^2^ test (i.e. contingency table), testing the null hypothesis that differences in CT and SA are equally distributed. Furthermore, a simulation strategy was used to assess whether the observed degree of overlap between differences in CT and SA is consistent with the idea of two spatially independent patterns. This hypothesis was tested based on N = 5000 randomly generated difference maps (i.e. maps containing random *t* values, thresholded at p < 0.05, two-tailed) for CT and SA. The extend of overlap (i.e. number of vertices with differences in CT and SA) was then assessed in each of the 5000 overlapping patterns to derive a probability value of obtaining a given percentage of overlap based on randomly varying patterns of differences. These statistical analyses were previously published by our group^11,21^.

### Data availability

Further details on the data and utilized software are available upon request from the corresponding author. The full set of raw data is not currently publicly available due to ethical restrictions. However, a subset of the sample can be made available upon request.

## Results

### Participant demographics and global brain measures

There were no significant differences in age or gender between 22q11.2DS and controls (*p* > 0.05, *two-tailed*; see Table 1), however, individuals with 22q11.2DS had a significantly lower full-scale IQ (*t*(117) = − 11.838, *p* < 0.001). Both groups were matched in terms of total cortical volume, and mean CT across the cortex. However, individuals with 22q11.2DS had a significantly reduced total surface area of the brain overall (*t*(117) = − 5.952, *p* < 0.001) (see Table 1 for details). A significant overall association was observed between IQ and total CV (*r* = 0.392, *p* < 0.001), as well as total SA (*r* = 429, *p* < 0.001), but not average CT (*r* = − 0.100, *p* = 0.278). These associations were also significant within the 22q11.2DS group (CV: *r* = 0.602, *p* < 0.001; SA: *r* = 0.400, *p* = 0.001), but not in the control group (CV: *r* = 0.185, *p* = 0.166; SA: *r* = 0.173, *p* = 0.194).

### Nested model comparison

Based on the nested model comparison, we established that the quadratic model provided a significantly better fit than the linear model in several clusters across the cortex when modelling neurodevelopmental trajectories for CV, CT and SA (see Supplementary Information S1). There were no brain regions where a significant improvement in fit was observed when the cubic term for age was included (RFT-based, cluster-corrected, *p* < 0.05) (see Supplementary Information S1). Thus, the quadratic model was chosen as the most parsimonious model for the examination of age-related differences in CV, CT and SA between individuals with 22q11.2DS and TD controls.

### Vertex-wise differences in CT and SA between 22q11.2DS and controls

**Figure 1:**
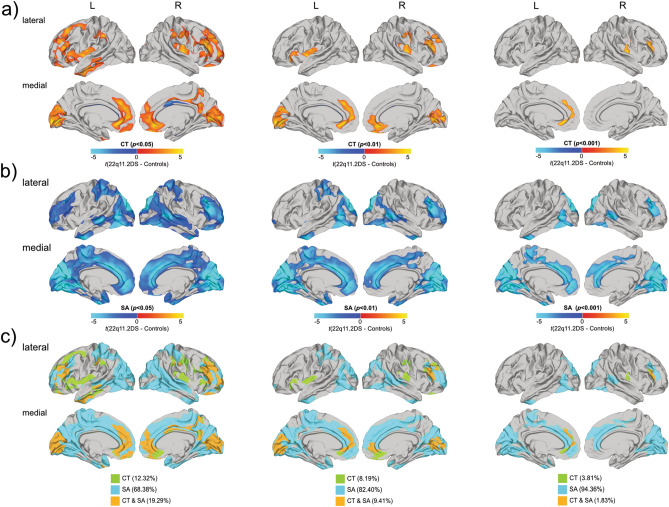
Significant differences in cortical thickness (**a**), surface area (**b**), and their overlap (**c**) in individuals with 22q11.2DS compared to controls. For (**a**) and (**b**), difference maps are displayed showing increased parameter estimates in 22q11.2DS in red to yellow, and decreased parameters in blue to cyan. For (**c**), vertex-wise differences in cortical thickness only are displayed in green, differences in surface area only are displayed in cyan, and overlapping differences in both cortical thickness and surface area are marked in orange. All figures (**a**–**c**) are presented at random-field-theory-based cluster-corrected (two-tailed) *p* < 0.05 (left panel), *p* < 0.01 (middle panel), and *p* < 0.001 (right panel).

Following correction for multiple comparisons (RFT-based cluster corrected, *p* < 0.05), individuals with 22q11.2DS relative to controls had significantly increased CT bilaterally in several spatially distributed clusters across the brain, including the dorsolateral-prefrontal cortices (DLPFC; approximate Brodmann area(s) [BA] 8–11/24/32/46–47), medial regions of the occipital lobe (BA 17–19), pre-and-post central gyrus (BA 1–5), supramarginal gyrus (BA 40), and left middle temporal gyrus (BA 20–21/32/38). Significant decreased CT was observed in a small bilateral cluster centered in cingulate gyrus (BA 24, 33) (see Fig. 1a, left panel). In addition, individuals with 22q11.2DS had significantly reduced SA in regions of the DLPFC (BA 8–11/23–33/46), the post central gyrus (BA 1–5), the cingulum (BA 23, 24), medial regions of the occipital lobe (BA 17–19), and temporal lobe (BA 20–21, 38) (Fig. 1b, left panel). See Supplementary Information S2 and S3 for details and cluster statistics. When applying a stricter cluster threshold (i.e. *p* < 0.01 and *p* < 0.001), the clusters with a significant between-group difference in CT and SA remained stable across thresholds in terms of their cluster peaks, although fewer clusters were observed at a the most conservative threshold of p < 0.001 for measures of CT (see Fig. 1a,b, middle and right panel). We did not observe any significant differences in CT and SA between 22q11.2DS individuals with and without prodromal symptoms of psychosis (see Supplementary Information S4).

**Figure 2:**
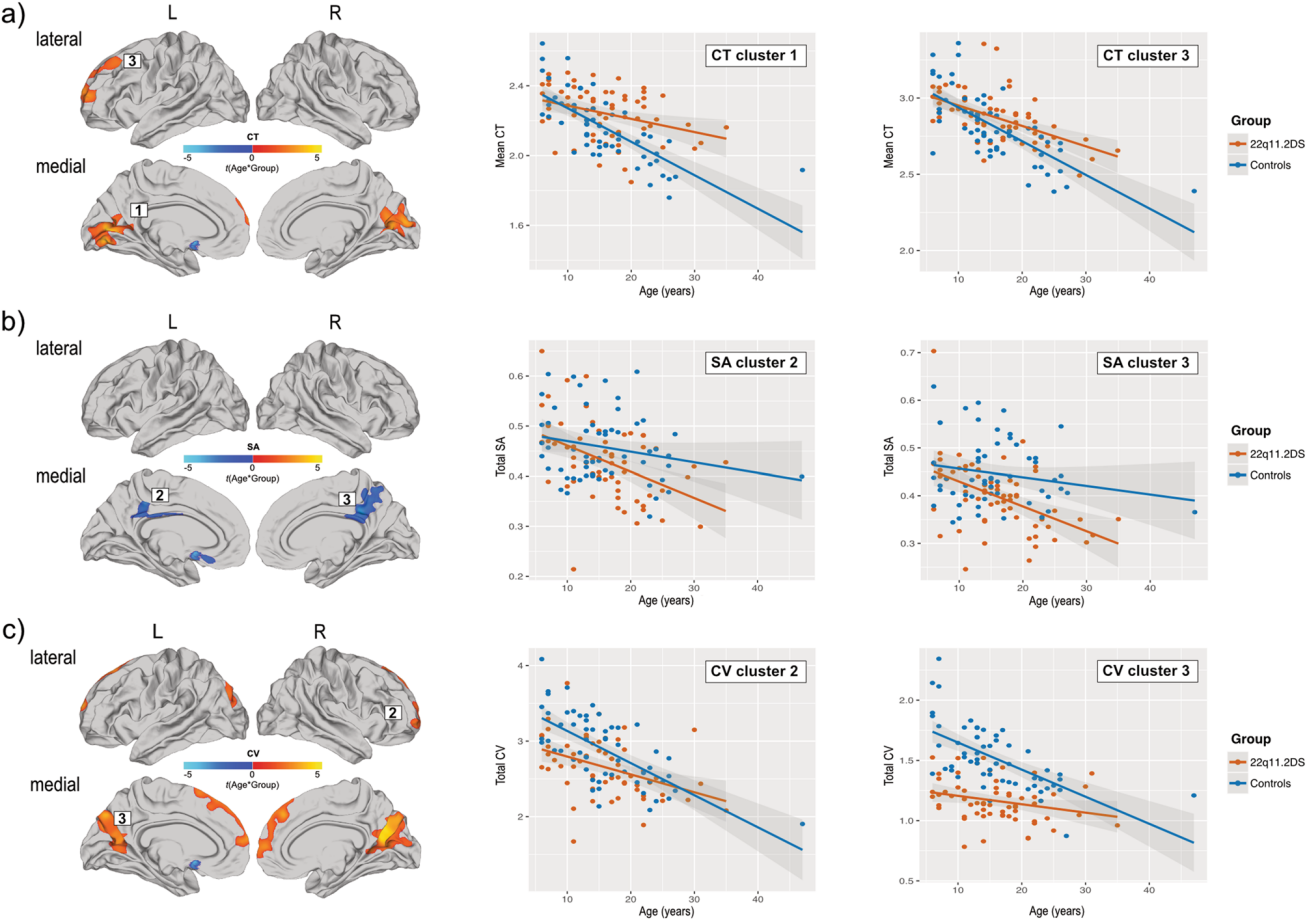
Developmental trajectories for CT (**a**), SA (**b**), and CV (**c**) in clusters with significant linear age-by-group interactions. Scatterplots and line of fit for 22q11.2DS are presented in Red, and in Blue for controls.

In several clusters with a significant between-group difference, we also observed that the cross-sectional neurodevelopmental trajectories for CT and SA significantly differed between groups as indicated by a significant linear age-by-group interaction. For example, for measures of CT, 22q11.2DS individuals display comparable values during early childhood relative to TD controls, with a less steep age-related decline during early adolescence in both the frontal as well as occipital cortex (see Fig. 2a) (see Supplementary Information S5 for details and cluster statistics). In contrast, for measures of SA, individuals with 22q11.2DS showed comparable values or no difference in SA in the left and right posterior cingulate cortex (respectively) during early childhood, followed by a steeper decline across age, with no intersecting growth curves, potentially due to the restricted age range examined (see Fig. 2b) (see Supplementary Information S5 for details and cluster statistics). There were no significantly clusters where we observe a significant age^2^-by-group interaction, which is why the interaction plots only depict the linear age-by-group interaction. Within these clusters, we did not observe any correlations with general cognitive abilities as measured by IQ indicating that covarying for IQ within the GLM was successful (see Supplementary Information S6).

### Spatial overlap of between-group differences in CT and SA

Across both hemispheres, at a statistical cluster-threshold of p < 0.05, we found that the largest proportion of all between-group differences in CT, SA, or both were caused by differences in SA (68.38%), while vertices with a significant difference in CT only explained about 12.32% overall. The number of vertices with a significant difference in SA thus significantly exceeded the number of vertices with a difference in CT by a factor of 5 (i.e. 68.38% vs. 12.32%, ${\chi }_{df=2}^{2}$= 38.94, *p* < 0.001). Furthermore, in 19.29% of all significantly different vertices, we found a difference in both CT and SA, i.e. the patterns of differences in CT and SA overlapped (see Fig. 1c, left panel and Table 2 for details). Simulations revealed that the probability of obtaining the same degree of overlap (i.e. 19.29%) between two randomly generated difference maps is very low (i.e. *p* = 0.0001). Thus, the observed percentage of overlap indicates that the majority of the differences in CT and SA in individuals with 22q11.2DS are not spatially independent (i.e. non-overlapping). As expected, when applying a stricter cluster threshold, we observe that the spatial overlap between CT and SA becomes less significant as the threshold becomes more conservative, with a larger effect sizes being attributed to measures of SA (see Fig. 1c).

**Table 2:** Spatial overlap between differences in cortical thickness and surface area.

Measure	No.^a^ (%)					
	Left hemisphere		Right hemisphere		Across hemispheres	
***p < 0.05***						
CT only	10,173	(11.04)	12,980	(13.56)	23,153	(12.32)
SA only	67,029	(72.74)	61,442	(64.19)	128,471	(68.38)
CT and SA	14,951	(16.22)	21,293	(22.25)	36,244	(19.29)
Total^b^	92,153	(100)	95,715	(100)	187,868	(100)
***p < 0.01***						
CT only	2604	(4.52)	6756	(11.90)	9360	(8.19)
SA only	50,061	(86.97)	44,139	(77.77)	94,200	(82.40)
CT and SA	4898	(8.51)	5860	(10.33)	10,758	(9.41)
Total^b^	57,563	(100)	56,755	(100)	114,318	(100)
***p < 0.001***						
CT only	529	(1.66)	1849	(6.08)	2378	(3.81)
SA only	30,876	(96.62)	27,977	(91.88)	58,853	(94.36)
CT and SA	552	(1.73)	590	(1.94)	1142	(1.83)
Total^b^	31,957	(100)	30,416	(100)	62,373	(100)

*CT *cortical thickness, *SA* surface area.

^a^Number of vertices displaying a significant group differences regardless of the sign.

^b^Total number of vertices with a significant difference in either CT or SA.

### Vertex-wise differences in CV between 22q11.2DS and controls

**Figure 3:**
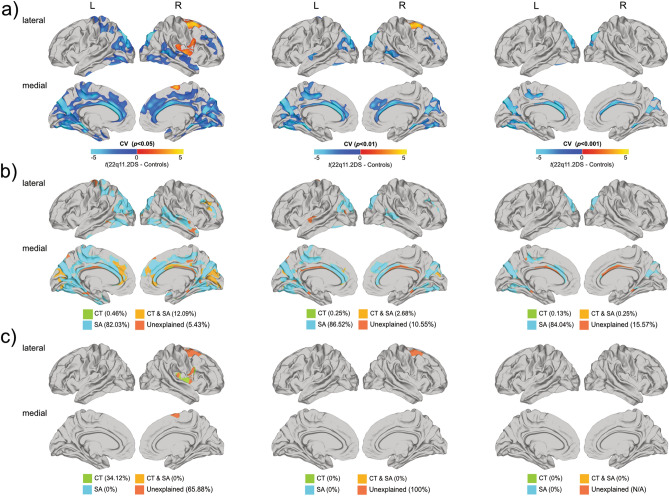
Significant differences in cortical volume between individuals with 22q11.2DS compared to controls (**a**), where increased parameter estimates in 22q11.2DS are marked in red to yellow, and decreases are marked in blue to cyan. Relative contributions of differences in cortical thickness and surface area to significant reductions in cortical volume are presented in (**b**), and increases in cortical volume in (**c**). For (**b**) and (**c**), vertex-wise contributions of cortical thickness are displayed in green, of surface area in cyan, and of a combination of both in orange. Volumetric differences unexplained by significant differences in cortical thickness or surface area are displayed in coral. All figures (**a**–**c**) are presented at random-field-theory-based cluster-corrected (two-tailed) *p* < 0.05 (left panel), *p* < 0.01 (middle panel), and *p* < 0.001 (right panel).

Following correction for multiple comparisons (*p* < 0.05) individuals with 22q11.2DS compared to controls, had significantly decreased CV in parieto-temporal regions (BA 17–20/30–31/37), cingulate cortices (BA 23–24), and in the occipital lobe (BA 17–18). Increased CV in 22q11.2DS was observed in right precentral and superior frontal (BA 4/6 (Fig. 3a, left panel) (see Supplementary Information S7 for cluster summaries and statistical details). As with CT and SA, when applying a stricter cluster threshold for CV, clusters with a significant between-group difference mostly remained stable across thresholds in terms of their cluster peaks, although fewer clusters were observed at a conservative threshold of *p* < 0.001 (see Fig. 3a, middle and right panel). There were no significant differences in CV between 22q11.2DS individuals with and without prodromal symptoms of psychosis (please see Supplementary Information S4).

We also observed significant age-by-group interactions for measures of CV in the lateral and medial prefrontal cortex, as well as in the medial occipital lobe (see Fig. 2c) (see Supplementary Information S5 for details and cluster statistics). Here, individuals with 22q11.2DS had reduced volume during early childhood and a less steep cross-sectional age-related decline in CV during adolescence and adulthood, with an intersecting growth curve in adulthood for the right medial frontal lobe. There were no significantly clusters with a significant age^2^-by-group interaction. Within the 22q11.2DS group, we also examined correlations between measures of IQ and CV in each significant cluster. However, IQ did not correlate significantly with any of these when correcting for multiple comparisons (*p* > 0.05) (see Supplementary Information S6).

### Contribution of differences in CT and SA to differences in regional CV

We subsequently examined vertices with a significant between-group differences in CV to identify whether the observed decreases in CV were driven by CT, SA or a combination of both (see Fig. 3). Following correction for multiple comparisons (*p* < 0.05), it was observed that the number of vertices with a difference in SA significantly exceeded the number of vertices with a difference in CT in terms of their relative contribution to overall differences in CV (82.03% vs. 0.46%; ${\chi }_{df=2}^{2}$ = 80.66, *p* < 0.001) (see Fig. 3b). Further, 12.09% of vertices were explained by differences in both CT and SA. The remaining 5.43% of significant reductions in CV could not be explained by significant differences in either SA or CT, and therefore seems be due to a combination of sub-threshold differences in both of these features (see Fig. 3b, left panel) (see Table 3 for details). In contrast, when we examined vertices with an increase in CV, we found that 34.12% of those overlapped with vertices showing a difference in CT only. The remaining 65.88% of volumetric differences, i.e. approximately two-thirds, could not be explained by a difference in either SA or CT. It is therefore likely that the differences in CV in these vertices are due to a combination of sub-threshold variations in both CT and/or SA (see Fig. 3c, left panel). When applying a stricter cluster threshold, the relative contribution of differences in CT and SA to volumetric differences shifted as the threshold becomes more conservative, with more vertices with a difference in CV being explained by differences in SA rather than CT (see Fig. 3b,c, middle and right panel) (see Table 3 for details).

**Table 3:** Relative contribution of differences in cortical thickness and surface area to differences in cortical volume.

	No.^a^ (%)					
	Left hemisphere		Right hemisphere		Across hemispheres	
***p < 0.05***						
**22q11DS < Control**						
CV	40,736	(100)	37,381	(100)	78,117	(100)
CT only	94	(0.23)	264	(0.74)	358	(0.46)
SA only	35,529	(87.22)	28,547	(76.37)	64,076	(82.03)
CT and SA	3059	(7.51)	6386	(17.08)	9445	(12.09)
CV explained	38,682	(94.6)	35,197	(94.19)	73,879	(94.57)
CV unexplained	2054	(5.04)	2184	(5.84)	4238	(5.43)
**22q11DS > Control**						
CV	0	0	6828	(100)	6828	(100)
CT only	0	(0)	2330	(34.12)	2330	(34.12)
SA only	0	(0)	0	(0)	0	(0)
CT and SA	0	(0)	0	(0)	0	(0)
CV explained	0	(0)	2330	(34.12)	2330	(34)
CV unexplained	0	(0)	4498	(65.88)	4498	(65.88)
***p < 0.01***						
**22q11DS < Control**						
CV	22,445	(100)	15,797	(100)	38,242	(100)
CT only	67	(0.33)	30	(0.16)	97	(0.25)
SA only	19,331	(86.13)	13,757	(87.09)	33,088	(86.52)
CT and SA	481	(2.14)	542	(3.43)	1023	(2.68)
CV explained	19,879	(88.57)	14,329	(90.67)	34,208	(89.45)
CV unexplained	2566	(11.43)	1468	(9.29)	4034	(10.55)
**22q11DS > Control**						
CV	0	(0)	1787	(100)	1787	(100)
CT only	0	(0)	0	(0)	0	(0)
SA only	0	(0)	0	(0)	0	(0)
CT and SA	0	(0)	0	(0)	0	(0)
CV explained	0	(0)	0	(0)	0	(0)
CV unexplained	0	(0)	1787	(100)	1787	(100)
***p < 0.001***						
**22q11DS < Control**						
CV	11,183	(100)	5429	(100)	16,612	(100)
CT only	22	(0.20)	0	(0.16)	22	(0.13)
SA only	9924	(88.74)	4037	(74.36)	13,961	(84.04)
CT and SA	42	(0.38)	0	(0)	42	(0.25)
CV explained	9988	(89.31)	4037	(74.36)	14,025	(84.43)
CV unexplained	1195	(11.43)	1392	(25.64)	2587	(15.57)
**22q11DS > Control**						
CV	0	(0)	0	(0)	0	(0)
CT only	0	(0)	0	(0)	0	(0)
SA only	0	(0)	0	(0)	0	(0)
CT and SA	0	(0)	0	(0)	0	(0)
CV explained	0	(0)	0	(0)	0	(0)
CV unexplained	n/a		n/a		n/a	

*CT* cortical thickness, *SA* surface area.

^a^Number of vertices displaying a significant group differences regardless of the sign.

^b^Total number of vertices with a significant difference in either CT or SA.

## Discussion

To our knowledge, this is the first study to quantify the overlap of vertex-wise differences in cortical thickness (CT) and surface area (SA) in individuals with 22q11.2DS compared to controls, as well as to identify their influence on between-group differences in cortical volume (CV). Our study confirms that individuals with 22q11.2DS have significantly increased CT and decreased SA in several large clusters across the cortex. These differences overlap in ~ 20% off all vertices. Moreover, we established that the increases in CV, which have been noted previously in 22q11.2DS, were primarily driven by increased CT, while decreases in CV were predominantly driven by commensurate decreases in SA. Our findings indicate that variability in CT and SA each contribute to volumetric differences in 22q11.2DS. However, their strong opposite effects in some areas of the brain mask a significant difference in CV. Thus, measures of CT and SA should be examined in isolation to better characterize the complex neuroanatomy of 22q11.2DS, and to guide future aetiological studies into the genetic underpinnings of atypical brain development in 22q11.2DS.

Differences in neuroanatomy associated with 22q11.2DS are well established, both globally and regionally^3,22^. Our results are largely consistent with previous studies examining measures of CT in 22q11.2DS. These mostly report spatially distributed increases CT in 22q11.2DS compared to controls, predominantly located in large parts of the frontal lobes, in the middle and inferior temporal lobes, and in the insula and medial occipital lobes^6–8^. Significant decreases in CT in 22q11.2DS were also reported by one study in the cingulate cortex^8^. Our findings are also consistent with previous studies examining vertex-wise estimates of SA, which seem to be mostly reduced in 22q11.2DS in the frontal, medial occipital and temporal lobes, and parts of the cingulate cortex^6–8^. Further, we observed several clusters where neurodevelopmental trajectories of CV, CT and SA differed significantly in 22q11.2DS. Taking together, these findings highlight that the 22q11.2 microdeletion causes widespread neuroanatomical abnormalities that might arise as a consequence of an atypical developmental trajectory of brain maturation^22^. Despite these extensive neuroanatomical abnormalities in a large number of brain regions, previous studies also note that some areas of the brain (e.g. the frontal lobes) seem to be relatively unaffected based on their grey matter volume, potentially as a consequence of diverging variability in CT and SA (i.e. an increase in CT and decreases in SA)^6,7^. This hypothesis has however never been tested directly.

In the present study, we therefore also compared the pattern of spatially distributed differences in CT with the pattern of difference in SA, as well as their contribution to differences in CV. We firstly observed that there were more differences in SA relative to CT overall, and that the number of vertices with a significant difference in SA exceeded the number of vertices with a significant different in CT by a factor of 5 (i.e. 68.38% vs. 12.32%). Moreover, many of the differences in CT and SA occurred in opposite directions, and also co-occurred in parts of the brain where we observed a significant difference in both features such as in the frontal lobe and medial occipital lobe. The frontal and occipital lobe thus seems to be particularly affected in 22q11.2DS as they are brain regions where differences in CT and SA converge. As such, neuroanatomical differences in these regions might be of particular relevance to 22q11.2, and contribute to the wide range of neuropsychiatric symptoms commonly observed in individuals with microdeletion. For example, the frontal lobe has previously been associated with social cognition, an aspect of mental functioning which individuals with 22q11.2DS often have great difficulties with^23,24^. Also, functional MRI studies show that individuals with 22q11.2DS have decreased frontal lobe activity across a variety of cognitive tasks (e.g. response inhibition, working memory, reward processing (reviewed in Ref.^25^), as well as disruptions in connectivity between the frontal lobe and the rest of the cortex^26^. Atypicalities in this regions have been linked to both ASD symptomatology^11^ and prodromal symptoms of psychosis in 22q11.2DS^26^. In the occipital lobe, volumetric alterations have been linked to more pronounced symptoms of psychosis in both 22q11.2DS and the general population^27,28^. Our findings, and those of others, therefore highlight that some areas of the brain seem to be more affected than others in terms of their neuroanatomy in 22q11.2DS. Yet, despite converging differences in CT and SA in this region, between-group differences in CT and SA were non-overlapping in the majority of vertices (i.e. ~ 80%), and as the threshold becomes more conservative, the spatial overlap between CT and SA becomes less significant, and should hence be considered to represent spatially independent patterns of variability. In the second part of our study, we therefore also focused on volumetric differences in the brain of individuals with 22q11.2DS and their relationship to variability in CT and SA.

Consistent with previous reports, when examining vertex-wise estimates of CV in isolation, we observed significantly reduced volume in the bilateral occipital and temporal cortices, and in the paracentral and cingulate cortex^6,8^. Also, in agreement with a previous study, we found a significant increase in CV in the bilateral pre-central cortex and the insula in 22q11.2DS^6^. Notably, while the frontal lobe presented a key area with converging differences in CT and SA, we did not observe any differences in the frontal lobe when examining measures of CV. Our results therefore suggest that independent variability in CT and SA may mask volumetric differences in some areas of the brain, which in turn appear to be relatively spared in volume by the microdeletion. Moreover, our study indicates that volumetric differences may be driven either by a difference in CT, SA or in both measures, and cannot unanimously be linked to a particular feature. More specifically, we found that 65% of all vertices with a difference in CV also showed a difference in CT, SA or a combination of both. Our data also suggests that overall reductions in CV are mainly driven by decreases in SA (82%) or by overlapping differences in CT and SA (12%). Overall increases in CV, however, were mostly due to increased CT, which explained about 34% of all volumetric increases in 22q11.2DS. When applying a more conservative cluster threshold, the relative contribution of differences in CT and SA to volumetric differences shifts, with more vertices being explained by differences in SA than CT. Last, we found that the remaining 66% of vertices with a difference in CV could not be explained by differences in either CT or SA directly, and are likely a result of subthreshold variability in these features. Taken together our findings and those of others, it is therefore vital to examine brain anatomy in 22q11.2 using multiple morphometric feature, and at a high degree of spatial resolution, to fully disentangle the neuroanatomical consequences of the microdeletion, and to link these to specific aspects of the pathophysiology of 22q11.2DS.

However, while there seems to be strong agreement with on the nature and the pattern of neuroanatomical differences observed in 22q11.2DS across studies, it is difficult to link the neuroanatomical differences observed in vivo to the particular genetic and molecular mechanisms underpinning 22q11.2. In terms of CT and SA, we know from neurobiological and genetic studies that CT is determined by the neuronal output from so called radial unit in the developing cortex, and it is thus related to the overall number of neurons within the cortical minicolumns^29^. By contrast, cortical SA is determined by the early proliferation of radial unit progenitor cells, and to the number of ontogenetic columns overall^29,30^. In the light of the current study, this means that our observed decrease of SA in 22q11.2DS might reflect reduced production of progenitor cells in multiple regions, originating early in cortical development, as suggested by previous studies^7,31^. In contrast, it has been suggested that the observed increased CT may occur as a result of over proliferation of intermediate progenitor cells during corticogenesi^6^. If so, the 22q11.2 deletion appear to be associated with both aberrant neural organisation and migration^8^, and a disruption of different genetic mechanisms in the 22q11.2 deletion at different developmental time points^6^. Further, there is recent evidence linking phenotypic differences to the size of the microdeletion, with individuals who have a larger deletion (i.e. low copy number repeats (LCRs) A-D) being more affected than those with a smaller deletion (LCRs A-B), which particularly applies to measures of SA^7^. Studies examining cortical grey matter in 22q11.2DS have suggested that genes located within the 22q11 microdeletion are essential for cortical formation, and thus partly the cause behind the aberrant cortical morphology observed in 22q11.2DS^31^. As such, the widespread alterations in SA and CT could provide important novel insight into neurobiological pathways that are key to atypical neurodevelopment in this population.

The independent associations of CT and SA more generally have previously been explored in both animal and human studies. For example, in rodent models, mutations of the genes *PAX6*, *LRP6*, and *NGN1/2* modify the abundance of intermediate progenitor cells and result in increases in CT but not SA^32^. In contrast, SA but not CT is modulated by variations in *MECP2* within specific cortical regions (e.g. cuneus and fusiform gyrus)^32^. A direct link between these genes and the neuroanatomical phenotype of 22q11.2DS remains to be established; however, there is preliminary evidence from both human and mice studies to suggest that several of the genes in the critical deleted region in 22q11.2 are expressed substantially in both the developing and adult brain, as well as in a broad range of other tissues^33^. It has been suggested that decreased cortical volume in 22q11.2DS is consistent with primary pathogenesis in the cortex during prenatal development^33^. Further, genetic studies in 22q11.2DS, both in animals and humans, have found several genes within the microdeletion site that are thought to be implicated in some of the psychiatric difficulties associated with 22q11.2DS. For example, *COMT* and *PRODH* have been linked to both psychosis and ASD^34,35^. Although there are only a few post-mortem studies in 22q11.2DS to date, which report subtle cortical lamination defects in a 3 month old^36^, and a loss or failure to differentiate upper layer projection neurones in the cortex^37^, these appear to relatively consistent with the changes in the cortical architecture that are typically observed in Schizophrenia, and to some extent ADHD and ASD^33^.

Studies examining cortical neuroanatomy in 22q11.2DS in relation to their psychiatric phenotype have also established a link between variability in CV, CT and SA and the individual’s clinical phenotype. For example, our group has recently reported that 22q11.2DS individuals with ASD symptomatology neuroanatomically differ from 22q11.2DS individuals without ASD, and from those with idiopathic ASD (i.e. ASD individuals without the microdeletion)^11,38,39^. In the present study we also examined CV, CT and SA between 22q11.2DS individuals with prodromal symptoms of psychosis compared to those without, and although we did not observe any differences potentially due to the small sample size, many previous studies have found that cortical regions implicated in idiopathic psychosis are also altered in 22q11.2DS. For example, alterations temporal and frontal regions have found to be predictive of positive symptoms in sub-threshold symptoms of psychosis^40,41^, prodromal symptoms of psychosis^42^, as well psychotic disorder^43^ in 22q11.2DS. Further, a recent large scale study by the ENIGMA consortium suggest that there are convergent mechanisms contributing to CT changes in those who develop psychosis, both in carriers and non-carriers of the microdeletion^7^. In the future, it will hence be important to consider different clinical subgroups of 22q11.2DS individuals when examine neuroanatomy based on different cortical features. Here, by examining neuroanatomical variability in individuals with 22q11.2DS using a set of specific anatomical features than go beyond traditional investigations into brain volume, we can start exploring the genetic and neurobiological mechanisms underpinning atypical brain anatomy in 22q11.2DS, and to link these to the somatic and psychiatric difficulties that often present in the condition. Furthermore, it will be crucial in the future to examine the shared and distinct mechanisms that mediate neuropsychiatric symptoms across disorders, e.g. idiopathic ASD and 22q11.2DS-related ASD and/or psychosis/schizophrenia, particularly when it comes to the development of biomarkers.

Our results need to be interpreted in the light of several methodological limitations. Firstly, we employed a multicentre design in order to obtain a larger sample size. However, the reliability of anatomical measures (such as CT and SA) has been shown to remain unaffected when MRI instrument and data processing factors are controlled for (Han et al. 2006). We therefore applied the same pre-processing pipeline and quality assessments to all our data. Further, due to difficulties assessing and scanning individuals with intellectual disability (IQ < 60), we only included those individuals who scored above 60 on the WASI, resulting in our 22q11.2DS individuals scoring around 10 points above average IQ found in the general 22q11.2DS population^44^. Further, we found an association between IQ and Group on overall (i.e. global) measures of CV, CT and SA. It is therefore important to control for IQ in the vertex-wise analysis, which we found effectively remove the confounding effect of IQ. Moreover, the control group included siblings of 22q11.2DS individuals and although this was controlled for in the statistical model, future studies would need to include a more matched comparison group. Moreover, while we did correct for age-effects in the statistical model, our sample included individuals from a wide age range (6–31 years). Given the fact that the brain development varies across different stages of development, it will be crucial to investigate replicate these results within well-defined age groups in the future. In general, the acquisition of large, well-matched longitudinal samples will be essential for further disentangling the neuroanatomical variability in this population, including examining the effects of deletion size on phenotypic differences. Last, our binary approach for examining the spatial overlap between differences in CT and SA, as well as their relative contribution to differences in CV, heavily relies upon the cluster-based significance threshold at which between-group differences are examined. As such, a lenient threshold that is better suited to examine the spatial overlap between patterns may in turn increase the occurrence of false positives. While we examined the results at different cluster thresholds (i.e. p < 0.05, 0.01, and 0.001), non-binary approaches that do not rely on a statistical threshold might be considered in the future to further characterize these patterns in 22q11.2DS.

Overall, our findings highlight that 22q11.2DS is associated with neuroanatomical alternations in CT and SA, potentially a consequence of an atypical developmental trajectory of brain maturation, as well as their distinct influence on measure of CV. The current study also demonstrates that neuroanatomical differences in 22q11.2DS are not limited to specific brain regions, but affect multiple cortical regions that are likely to impact on the various clinical phenotypes associated with 22q11.2DS.

## Supplementary information

Supplementary Information.
