# Supplementary material for: Brain morphometry in 22q11.2 deletion syndrome: an exploration of differences in cortical thickness, surface area, and their contribution to cortical volume
Source: Sci Rep. 2020 Nov 2;10:18845. doi: 10.1038/s41598-020-75811-1 (PMC7606591; doi:10.1038/s41598-020-75811-1)
Supplement: Supplementary file 1 — Supplementary Information. [file 41598_2020_75811_MOESM1_ESM.pdf]

Supplementary Materials

Title: Brain Morphometry in 22q11.2 Deletion Syndrome: An exploration of differences in cortical thickness, surface area, and their contribution to cortical volume.

Authors: M. Gudbrandsen MSc[a], E. Daly PhD[a], C.M. Murphy PhD[a,b], C.E. Blackmore MSc[a,b], M. Rogdaki PhD[c,d,], C. Mann PhD[e], A. Bletsch PhD[e], L. Kushan MSc[f], C.E. Bearden PhD[f], D.G.M. Murphy PhD[a], M.C. Craig PhD[a,g] & C. Ecker PhD[e]

S1 – Nested model comparison of Age effects 2
S2 – Cortical Thickness clusters of significant differences in 22q11.2DS compared to controls.. 3
S3 – Cortical Thickness and Surface Area clusters of significant differences in 22q11.2DS compared to controls 4
S4 – Subgroup analysis of prodromal symptoms of psychosis 5
S5 – Cortical volume, cortical thickness and surface areas clusters with significant group-by-age linear interactions 6
S6 – Group-by-IQ associations 7
S7 – Cortical Volume clusters of significant differences in 22q11.2DS compared to controls...... 8

S1 – Nested model comparison of Age effects

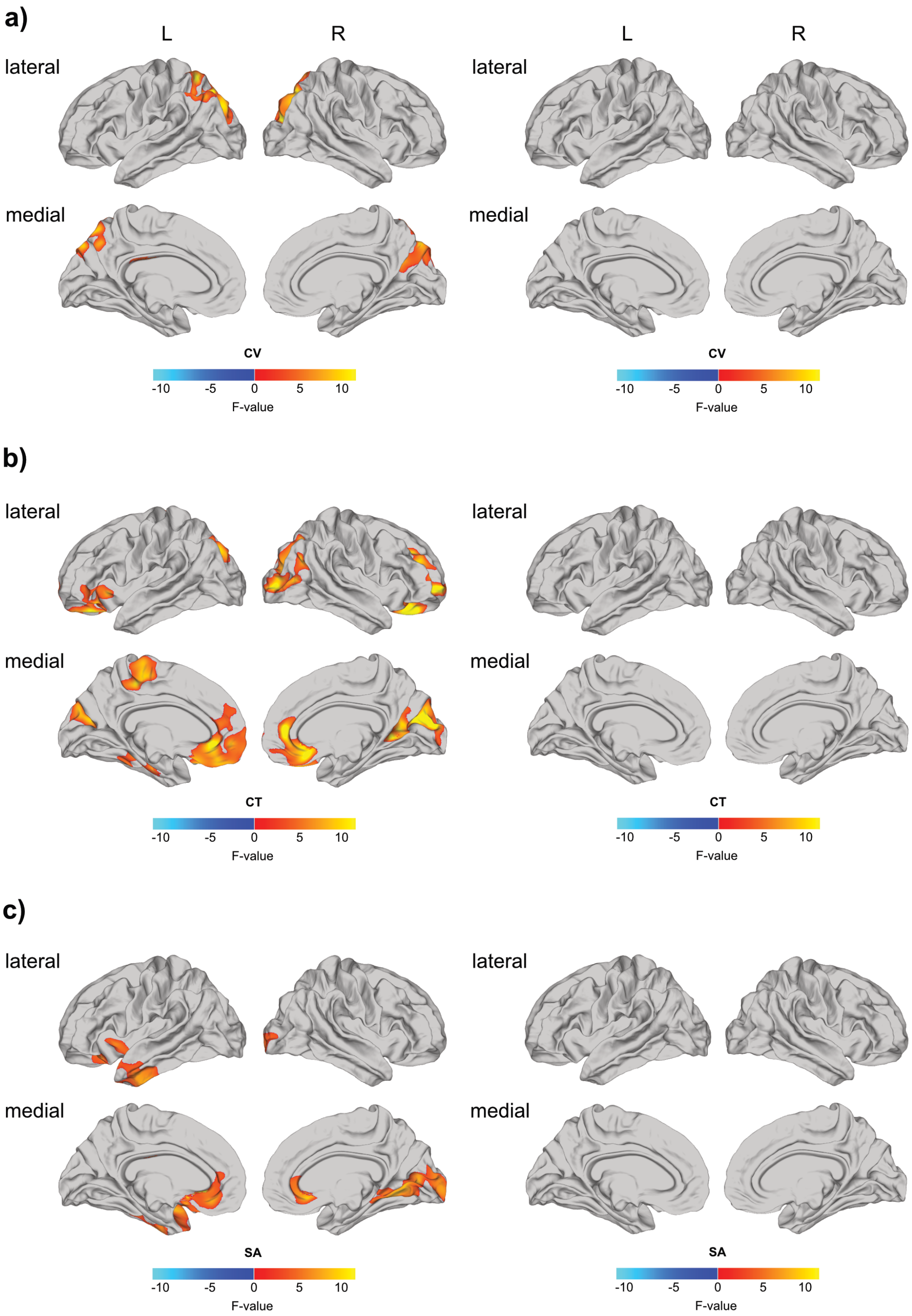

Nested model comparisons for (a) cortical volume (CV), (b) cortical thickness (CT), and (c) surface area (SA). The right panel shows the comparison of the linear vs. quadratic model, and the left panel shows the quadratic model vs. cubic model, including all age effects and age-by-group interactions. *F*-values (blue to red) indicate voxels where the more complex model provided a better fit than the more reduced model (*F*-statistic, thresholded). Model comparisons are indicated by random-field theory (RFT)-based, cluster-corrected ($p<0.05$) difference maps in goodness of fit (based on *F*-statistics).

S2 – Cortical Thickness clusters of significant differences in 22q11.2DS compared to controls

Contrast	Cluster	Region Labels	Hemisphere	BA	Vertices	Talairach x	Talairach y	Talairach z	t_{max}	$p_{cluster}$
CT										
Case > Control										
	1	Caudal middle frontal gyrus, lateral orbital frontal cortex, medial oribitofrontal cortex, pars orbitalis, pars triangularis, precentral gyrus, rostral anterior cingulate cortex, rostral middle frontal gyrus, superior frontal gyrus	R	8-11, 24, 32, 46-47	14612	10	25	-16	4.40	1.1×10^{-5}
	2	Lateral orbital frontal cortex, medial oribitofrontal cortex, rostral anterior cingulate gyrus, superior frontal gyrus	L	8-11, 24, 32, 43-47	8442	-7	33	-6	4.46	1.1×10^{-5}
	3	Postcentral gyrus, precentral gyrus, supramarginal gyrus	R	1-4, 40	6063	42	-12	29	4.43	1.8×10^{-5}
	4	Cuneus cortex, lateral occipital cortex, lingual gyrus & pericalcarine cortex	R	17-19	4676	6	-72	6	4.34	2.0×10^{-5}
	5	Rostral middle frontal gyrus, superior frontal gyrus	L	4, 6, 8-9	3959	-22	39	20	3.72	2.4×10^{-5}
	6	Cuneus cortex, lateral occipital cortex, lingual gyrus & pericalcarine cortex	L	17 - 19	4433	-5	-81	3	4.52	3.5×10^{-5}
	7	Isthmus-cingulate cortex, posterior-cingulate cortex & precuneus cortex	R	4, 6, 32-33, 44, 52	4149	7	-19	37	3.68	5.1×10^{-5}
	8	Inferior temporal gyrus, middle temporal gyrus, temporal pole	L	20-21, 32-	3303	-53	-8	-23	4.82	1.9×10^{-4}
	9	Insula, postcentral gyrus, supramarginal gyrus	R	40	3866	36	-14	7	4.23	1.0×10^{-3}
	10	Postcentral gyrus, supramarginal gyrus	L	10, 46	2714	-56	-38	36	4.06	3.8×10^{-3}
	11	Rostral middle frontal gyrus	L	46	2311	-35	35	12	3.83	4.4×10^{-3}
Case < Control										
	12	Caudal anterior-cingulate cortex	L	24	338	-1	21	16	-5.00	1.0×10^{-5}
	13	Caudal anterior-cingulate cortex, posterior-cingulate cortex	R	33	872	3	9	24	-3.62	1.0×10^{-5}
	14	Uncus	R	28	84	25	-9	-25	-3.14	1.6×10^{-5}

Hemisphere: *L* : Left, *R* : Right; *BA* : approxminate Brodmann area at t_{max}; vertices: number of vertices within the cluster; tmax; maxiumum t-statistic within the cluster; Pcluster: cluster-corrected p-value.

S3 – Cortical Thickness and Surface Area clusters of significant differences in 22q11.2DS compared to controls

Contrast	Cluster	Region Labels	Hemisphere	BA	Vertices	Talairach x	Talairach y	Talairach z	t_{max}	$p_{cluster}$
SA										
Case < Control										
	1	Banks superior temporal sulcus, caudal anterior-cingulate cortex, caudal middle frontal gyrus, cuneus cortex, entorhinal cortex, frontal pole, fusiform gyrus, inferior parietal cortex, inferior temporal gyrus, isthmus-cingulate cortex, lateral occipital cortex, lateral orbital frontal cortex, lingual gyrus, medial orbital frontal cortex, middle temporal gyrus, paracentrale lobule, parahippocampal gyrus, pars opercularis, pars triangularis, pericalcarine cortex, postcentral gyrus, precuneus cortex, rostral anterior cingulate gyrus, rostral middle frontal gyrus, superior frontal gyrus, superior parietal cortex, superior temporal gyrus, temporal pole, transverse temporal cotex.	L	1-7, 9-11, 17, 19-24, 32-33, 37-38, 40, 46	81708	-15	-65	15	-7.12	4.9×10^{-6}
	2	Banks superior temporal sulcus, caudal anterior-cingulate cortex, caudal middle frontal gyrus, cuneus cortex, entorhinal cortex, frontal pole, fusiform gyrus, inferior parietal cortex, inferior temporal gyrus, isthmus-cingulate cortex, lateral occipital cortex, lateral orbital frontal cortex, lingual gyrus, medial orbital frontal cortex, middle temporal gyrus, paracentrale lobule, parahippocampal gyrus, pars triangularis, pericalcarine cortex, postcentral gyrus, posterior-cingulate cortex, precentral gyrus, precuneus cortex, rostral anterior cingulate gyrus, rostral middle frontal gyrus, superior frontal gyrus, superior parietal cortex, superior temporal gyrus, supramarginal gyrus, temporal pole, transverse temporal cotex.	R	1-3, 17, 19-24, 28, 37-38, 40, 45-46	82590	14	-77	15	-7.30	4.9×10^{-6}
	3	Posterior-cingulate cortex	L	24	47	-2	-7	27	-3.27	2.8×10^{-2}
	4	Posterior-cingulate cortex	R	24	40	3	7	25	-3.64	3.2×10^{-2}

Hemisphere: *L* : Left, *R* : Right; *BA* : approxminate Brodmann area at t_{max}; vertices: number of vertices within the cluster; tmax; maxiumum t-statistic within the cluster; Pcluster: cluster-corrected p-value.

S4 – Subgroup analysis of prodromal symptoms of psychosis

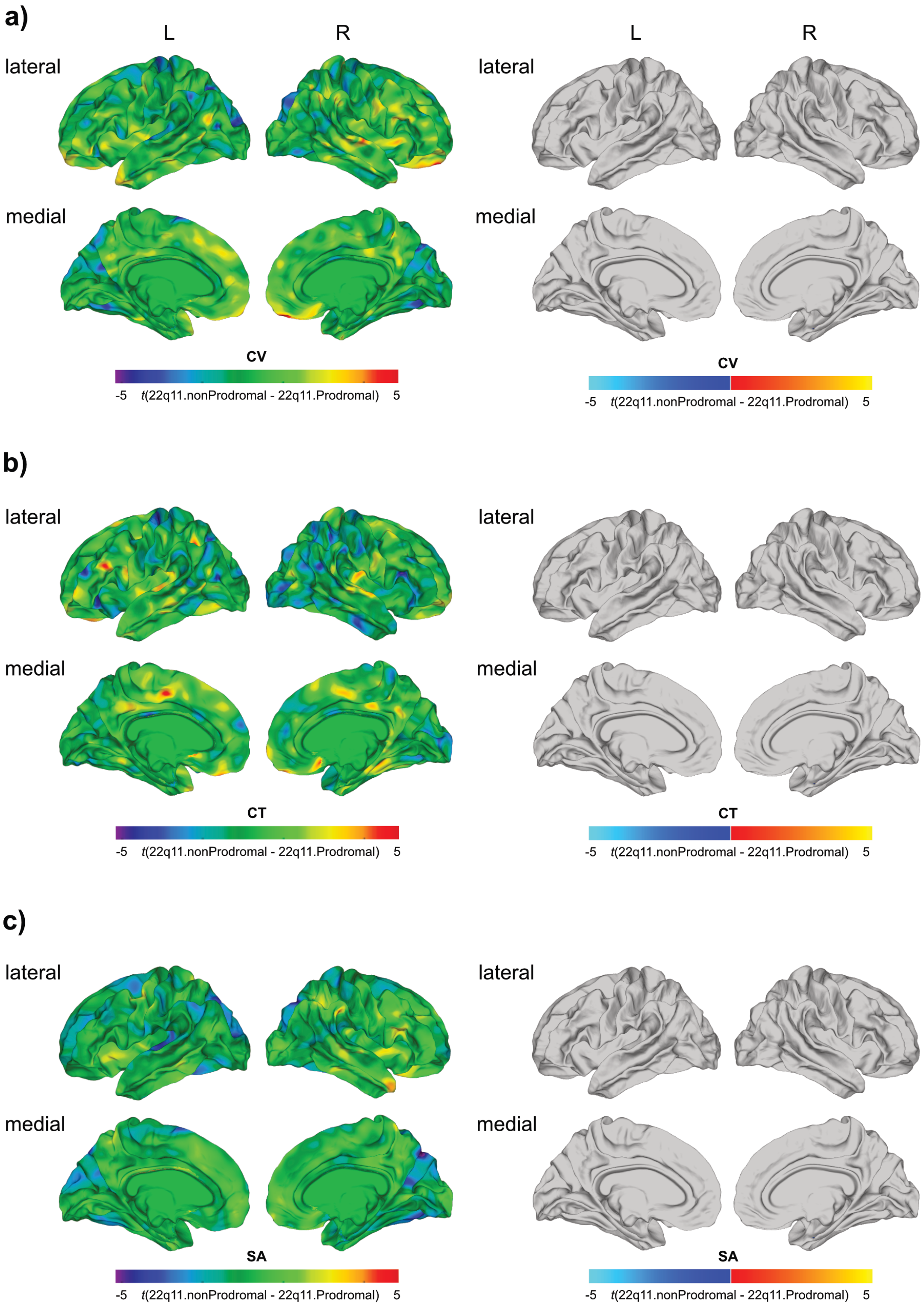

Significant differences in (a) cortical volume (CV), (b) cortical thickness (CT), and (c) surface area (SA) in individuals with 22q11.2DS and no prodromal symptoms of psychosis (22q11.nonProdromal), compared to individuals with 22q11.2DS and prodromal symptoms of psychosis (22q11. Prodromal). The left panel shows the un-thresholded *t*-maps, where increases in 22q11.nonProdromal relative to 22q11.Psychosis are indicated in yellow to red, and decreases in blue to purple. The right panel shows random-field-theory (RFT)-based cluster-corrected (p<0.05, two-tailed) difference maps. Following correction for multiple comparisons, there were no significant group differences between 22q11.nonProdromal and 22q11. Prodromal in any of the examined cortical descriptors.

S5 – Cortical volume, cortical thickness and surface areas clusters with significant group-by-age linear interactions

Contrast	Cluster	Region Labels	Hemisphere	BA	Vertices	Talairach			t_{max}	$p_{cluster}$
						x	y	z		
CV										
	1	Cuneus cortex, pericalcarine cortex, precuneus cortex, superior parietal cortex	R	7, 18, 31	4819	22	-61	26	4.48	4.7×10^{-4}
	2	Superior frontal gyrus	R	8-10	2081	9	38	35	3.32	5.0×10^{-3}
	3	Cuneus cortex, pericalcarine cortex, precuneus cortex, superior parietal cortex	L	7, 31	3917	-17	-73	38	3.56	5.2×10^{-3}
	4	Superior Frontal cortex	L	6, 8-10	1786	-11	60	13	3.33	4.0×10^{-2}
	5	Medial orbitofrontal cortex	L	30	109	-5	8	-7	-4.02	1.3×10^{-2}
CT										
	1	Lingual gyrus, pericalcerine cortex	L	17-18	3347	-20	-73	7	4.18	1.8×10^{-3}
	2	Cuneus cortex, pericalcarine cortex	R	17-18	2477	23	-67	6	3.78	7.5×10^{-3}
	3	Rostral middle frontal gyrus, superior frontal gyrus	L	8-10	2082	-25	15	38	3.35	1.1×10^{-2}
	4	Medial orbitofrontal cortex	L	30	87	-5	9	-7	-4.14	6.4×10^{-3}
SA										
	1	Medial orbitofrontal cortex	L	30	280	-6	8	-8	-3.96	3.7×10^{-3}
	2	Precuneus, posterior cingulate cortex	L	23, 31	1311	-7	-36	39	-2.81	99×10^{-3}
	3	Precuneus, posterior cingulate cortex	R	23, 31	3194	11	-39	32	-3.45	1.1×10^{-2}

Hemisphere: *L* : Left, *R* : Right; *BA* : approxminate Brodmann area at t_{max}; vertices: number of vertices within the cluster; tmax; maxiumum t-statistic within the cluster; Pcluster: cluster-corrected p-value.

S6 – Group-by-IQ associations

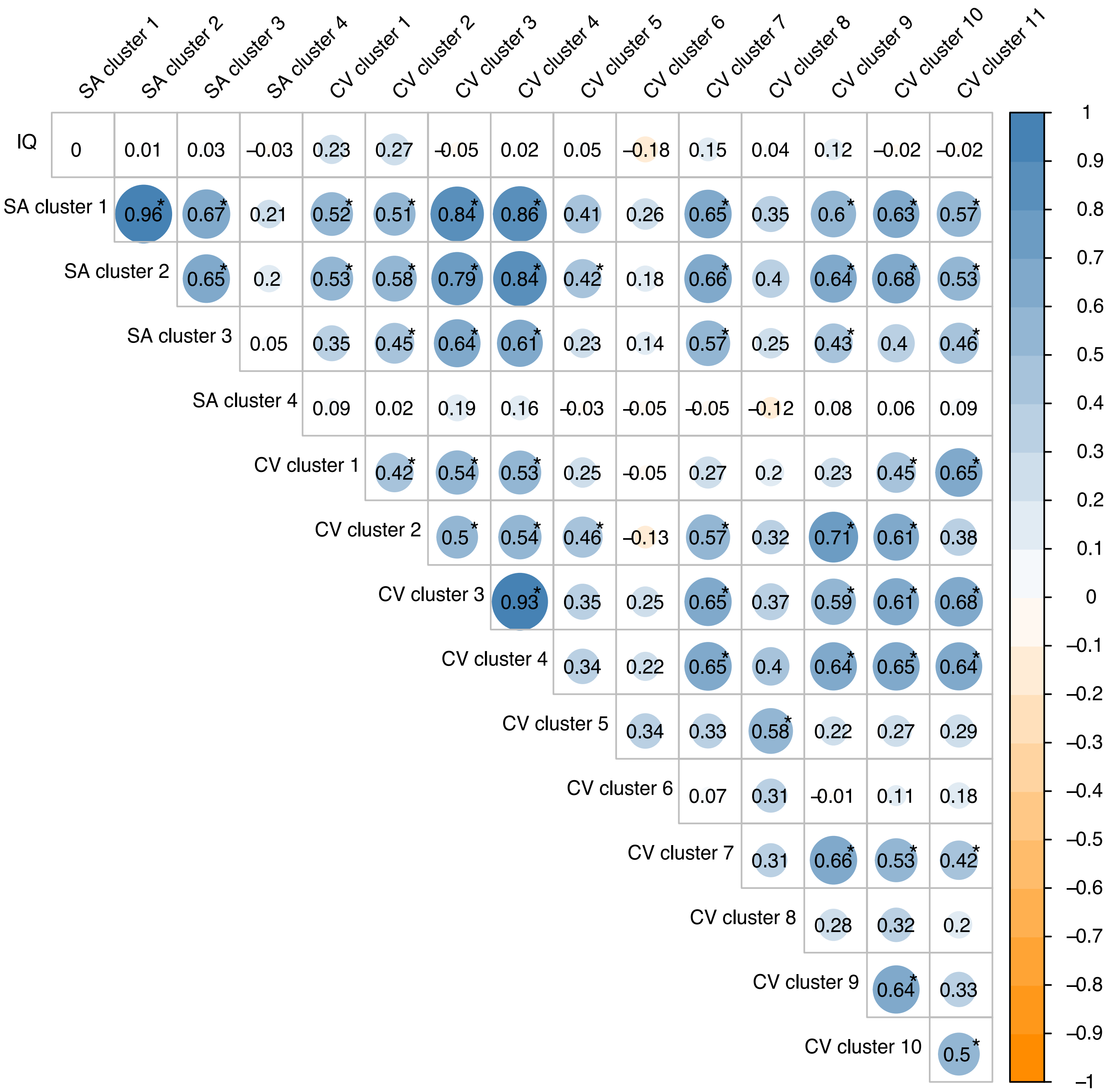

Correlations between IQ and clusters with significant group difference in cortical volume (CV) and surface area (SA) between individuals with 22q11.DS and typically developing controls. Positive correlations are indicated in blue, negative correlations are indicated in orange. Significant correlations ($p < 0.05$, Bonferroni corrected) are highlighted with *.

S7 – Cortical Volume clusters of significant differences in 22q11.2DS compared to controls

Contrast	Cluster	Region Labels	Hemisphere	BA	Vertices	Talairach x	Talairach y	Talairach z	t_{max}	$p_{cluster}$
CV										
Case > Control										
	1	Precentral gyrus, superior frontal gyrus	R	4, 6	3418	20	-5	53	4.36	2.2×10^{-3}
Case < Control										
	2	Caudal anterior-cingulate cotex, cuneus cortex, fusiform gyrus,	L	4-5, 7, 9,	37968	-22	-43	-4	-7.23	8.2×10^{-6}
	3	Cuneus cortex, fusiform gyrus, inferior parietal cortex, inferior temporal gyrus, isthmus-cingulate cortex, lateral occipital	R	17-20, 30-31, 37	18695	24	-58	21	-5.50	8.2×10^{-6}
	4	Caudal anterior-cingulate cotex, isthmus-cingulate cortex, posterior-cingulate cortex, rostral anterior cingulate cortex,	R	8-9, 23-24, 32	6649	6	6	29	-5.09	8.2×10^{-6}
	5	Entorhinal cortex, fusiform gyrus, inferior temporal gyrus	L	20, 37	1922	-36	-30	-17	-4.87	9.4×10^{-6}
	6	Uncus	R	28	118	25	-9	-25	-3.13	1.2×10^{-5}
	7	Uncus	L	28	495	-30	-1	-24	-3.32	4.2×10^{-4}
	8	Rostral middle frontal gyrus	R	9, 46	3034	3	37	18	-4.00	4.2×10^{-4}
	9	Superior temporal gyrus, transverse temporal cortex	R	22, 42, 38	3482	47	10	-22	-3.40	2.1×10^{-3}
	10	Banks superior temporal sulcus, inferior parietal cortex, middle temporal gyrus	R	39-40	3309	57	-53	8	-4.12	5.2×10^{-3}
	11	Paracental lobule, superior frontal gyrus	R	1-4, 24	2379	15	-14	40	-3.14	2.3×10^{-2}

Hemisphere: L: Left, R: Right; BA: approxminate Brodmann area at tmax; vertices: number of vertices within the cluster; tmax; maxiumum t-statistic within the cluster; Pcluster: cluster-corrected p-value.A2:L32
